# The Obstructive Lung Diseases Program: Integrated obstructive lung disease services within primary care in Pakistan

**DOI:** 10.12669/pjms.38.ICON-2022.5781

**Published:** 2022-01

**Authors:** Saima Saeed, Madiha Siddiqui, Rahma Altaf

**Affiliations:** 1 Dr. Saima Saeed, MRCP (Resp Med) (UK), MSc, The Indus Hospital & Health Network, Karachi, Pakistan; 2 Dr. Madiha Siddiqui (FCPS, Pulmonology), The Indus Hospital & Health Network, Karachi, Pakistan; 3 Rahma Altaf (BSc Economics), The Indus Hospital & Health Network, Karachi, Pakistan

**Keywords:** Obstructive lung disease, E-curriculums, Integrated care program, Spirometry

## Abstract

**Objective:**

To assess the learning impact of e-curriculums on healthcare professionals (HCPs). The second objective was to report the screening, detection and clinical features of patients with obstructive lung diseases (OLD) through an integrated care program at The Indus Hospital & Health Network (IHHN), Karachi, Pakistan.

**Methods:**

A retrospective, observational study was conducted in the Family Medicine outpatient department from January 2019 till July 2021. HCPs were trained on the diagnosis and management of OLD through e-learning. Patients were screened clinically for OLD and had spirometry performed if suspect. Baseline characteristics, patient-reported outcome measures (PROMs), spirometry and treatment modalities were collected. Univariate analysis was done on Excel and paired t-testing was performed on Stata 16.

**Results:**

Online training on clinical aspects of OLD was completed by 33 HCPs, amongst whom 77.9% demonstrated improved post-test evaluations of 26.8% (p=0.000). Of 1896 patients screened, 60.8% were diagnosed as OLD. Asthmatics accounted for 66.5% (60.9% females, median age 39 years). In 84.5% of patients who completed PROMs, poor control of symptoms was reported. Inhaler technique was taught in 66.5%. Breathless patients, with a high modified Medical Research Council score (mMRC ≥ 2, n=234), were referred for pulmonary rehabilitation in 92% of cases. Tobacco cessation advice was delivered to 61.1% of all current users (n=229).

**Conclusion:**

The OLD program uses capacity building, gold standard diagnostics and updated management strategies in primary care, allowing earlier diagnosis of suspected patients and implementation of evidence-based interventions, aiming to improve their morbidity and mortality.

## INTRODUCTION

Non-communicable diseases (NCD) are gaining recognition globally, and are a major public health challenge. In a ten-year review, the World Health Organization (WHO) declared chronic respiratory diseases (CRD), affecting the lungs and airways, one of the four most detrimental NCDs.^1^ These include Obstructive Lung Diseases (OLD), namely Chronic Obstructive Pulmonary Disease (COPD) and asthma, prevalent worldwide.^2^ Generally, clinical presentation is with breathlessness, cough, and wheeze. Breathlessness is scored using four-point, modified Medical Research Council (mMRC) score which increases with intensity of dyspnea. Cumulative exposure to tobacco and biomass can contribute to both the pathogenesis (especially of COPD) and acceleration of disease. Spirometry is the diagnostic test of choice and is performed to detect airflow obstruction. Patient-reported outcome measures include Asthma Control Test (ACT) and COPD Assessment Test (CAT), documenting symptom control. Treatment focuses on inhaled bronchodilators and steroid therapy, which may be difficult for patients to use correctly. Inadequate therapy and poor technique may lead to disease flare ups (“exacerbations”) requiring emergency care. Optimizing treatment type and technique, and interventions such as tobacco cessation advice, prophylactic vaccination against pneumococcus and influenza, risk factor avoidance and pulmonary rehabilitation (PR) are highlighted in international guidelines for the management of asthma (GINA)^3^ and COPD (GOLD).^4^ These evidence-based strategies are designed to address the morbidity and mortality of these CRDs.

In low-middle-income countries (LMICs), the prevalence of COPD and asthma cannot be fully understood due to underdiagnosis,^5^ yet a recent systemic review showed over 80% of deaths were attributed to them^6^. In South Asia, these diseases accounted for 8% of all Disability–Adjusted Life Years.^7^ The need for increasing general awareness about COPD and access to diagnostics has been highlighted in LMICs.^8^ Careful estimates of the prevalence of COPD and asthma in Pakistan are 2.1% and 4.3% respectively.^9^ With 61.4% of the Pakistani population residing in rural areas, the lack of strong primary care healthcare systems leads to reduced accessibility to health facilities for vulnerable populations.^10^ The most recent local paper dates back to 2011 where adherence to asthma guidelines was suboptimal amongst Karachi general practitioners.^11^ Additionally, 20% of Pakistani adults smoke tobacco^12^ and there is increased tobacco consumption amongst the youth.^13^ Indoor and outdoor biomass exposure such as wood burning stoves leads to airflow limitation and development of comorbid conditions like lung cancer.^14^ Spirometry, inhaler technique review, patient counselling and PR programs are also not widely available.^15^

Only 20% of the estimated asthma and COPD cases are currently being identified and diagnosed in primary care in Pakistan.^15^ This issue may be addressed using an integrated, multidisciplinary approach as seen in integrated practice units (IPU) and integrated care programs (ICP). IPUs provide comprehensive services for a medical condition using a multidisciplinary approach. An ICP offers seamless care across multiple health and non-health sectors to optimize patient-centric outcomes. Indus Hospital and Health Network (IHHN) has an expanding primary care program with 25 primary care facilities and 12 standalone clinics nationwide.^16^ Opportunities thus exist for targeted capacity building and the integration of lung health services at the patients first port of call, coordinating and unifying the approach to these common CRDs.

## METHODS

### Training:

An e-learning platform, Canvas, was used to train relevant healthcare professionals (HCP) in four modules: spirometry, COPD, asthma and inhalers. Bespoke modules were developed based on internationally accepted guidelines.

Each module consisted of a (1) thirty-minute pre-course assessment, (2) overview, (3) main objectives, (4) marking criteria, (5) course content and (6) thirty-minute post-course assessment. Illustrations, algorithms and quizzes were added to enhance the course content.

Both pre- and post-course assessments involved ten questions in a case-based format. Marking criteria stipulated scores greater than 70% as passing and eligible for completion certificates while lower scores are offered a re-attempt option to be completed within the same week.

The module learning cycle had a four-week format:

- **Week zero:** Pre-course assessment,
- **Week one and two:** HCPs read through course content,
- **Week three:** Post-course assessment,
- **Week four**: Pre- and post-course assessment results compiled and distributed; re-attempts offered where necessary.

Learning was supported by online and selected in-person interactive sessions where needed. Nurses further shadowed Consultant Pulmonologists in outpatients to become Specialist Lung Health nurses.

### Study Design:

A retrospective, observational study was conducted at the Family Medicine outpatient department at The Indus Hospital – Korangi campus from January 2019 to July 2021. Patients with breathlessness, wheeze, cough and other clinical features suggestive of OLD were referred for evaluation. Characteristics including demographics, occupation, comorbidities, biomass exposure and tobacco status were captured. Spirometric data with reversibility was used to diagnose asthma and COPD and to assess severity of obstruction using Forced Expiratory Volume in first second (FEV1) values. Patient-reported outcome measurements were used to assess control of disease, CAT ≥ 10 and ACT ≤ 19 showing poor control (Table 1). Numbers of annual exacerbations of disease and mMRC were also recorded. These variables were used to derive the GOLD staging in COPD. Management strategies were identified using frequency of medications, inhaler technique, tobacco cessation advice and percentage of PR referrals.

**Table I:** Asthma Control and COPD Assessment Tests.

Asthma Control Test (ACT): Each question is scored from 1 to 5 points, where 1 is the worst score (most frequent)	
Q1	Activity level affected at work, school or home
Q2	Shortness of breath
Q3	Sleep quality
Q4	Reliever medication frequency
Q5	Asthma control perception

***COPD Assessment Test (CAT): Each question is scored out from 0 to 4 points, where 5 is the worst score (always present/impaired)***	

Q1	Cough
Q2	Phlegm
Q3	Chest tightness
Q4	Shortness of breath
Q5	Activity level
Q6	Ease of leaving home
Q7	Sleep quality
Q8	Energy level

### Analysis:

An electronic data collection tool, REDCap, was used for data collection. Excel was used for univariate analysis. Quantitative data was expressed as percentage and median (interquartile range (IQR)) where appropriate. Stata 16 was used for statistical testing (paired t-test) with significance set at p<0.01. Ethical approval was obtained from the Institutional Review Board IRD_IRB_2020_05_013 and IRD_IRB_2021_05_021.

## RESULTS

### Training:

**Fig.1:**
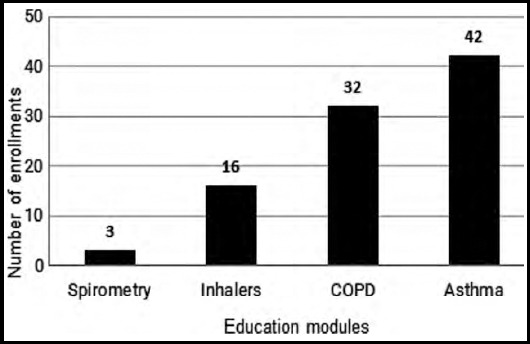
Total number of enrollments per module.

A total of 33 HCPs (30 primary care physicians and three nurses) enrolled in the online training modules (Fig.1). Each physician took the inhalers, COPD and asthma course and three nurses took spirometry courses. When comparing cumulative pre- and post-course assessment scores, 77.9% showed an improvement in knowledge scores by 26.8% (99% CI; 0.99-2.36; p=0.000).

### Baseline characteristics:

**Fig.2:**
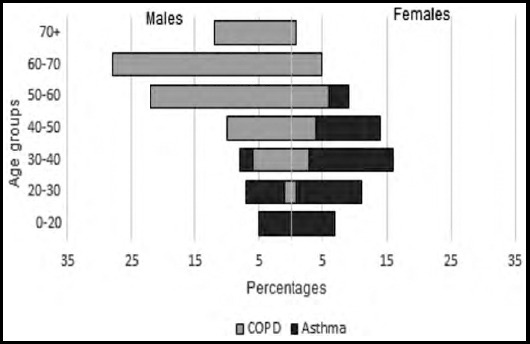
Population pyramid for obstructive lung diseases.

Since 2019, a total of 1896 patients were screened on first visit. Of these, 60.8% were diagnosed as OLD on both spirometric and/or clinical grounds. Asthma (66.5%, n=767) was more common in females (60.8%) with a median (IQR) age of 39 (28-50) years and COPD (33.5%, n=387) in males (79.1%) with a median (IQR) age of 60 (50-67) years (Fig.2). By occupation, most patients (n=96) worked in textile (12.5%), construction (8.3%) and transportation (6.3%). Comorbidities were present in 26.3% with hypertension (17.4%), diabetes (10.2%) and ischemic heart disease (1.6%) being common. Comorbidities were more prevalent in asthmatic patients (55.9%). A history of burnt biomass exposure (wooden stoves, 14.2% and fumes, 5%) was seen in asthma (24.9%) and COPD (29.2%) with median (IQR) exposure of 15 (10-25) years. Tobacco use was identified in 479 patients whilst 50.9% were current users. Of these, 34% were smokers and 16.9% consumed smokeless tobacco. Smoking history was reported in 22% of asthmatics.

### Diagnostics and PROMs:

**Fig.3:**
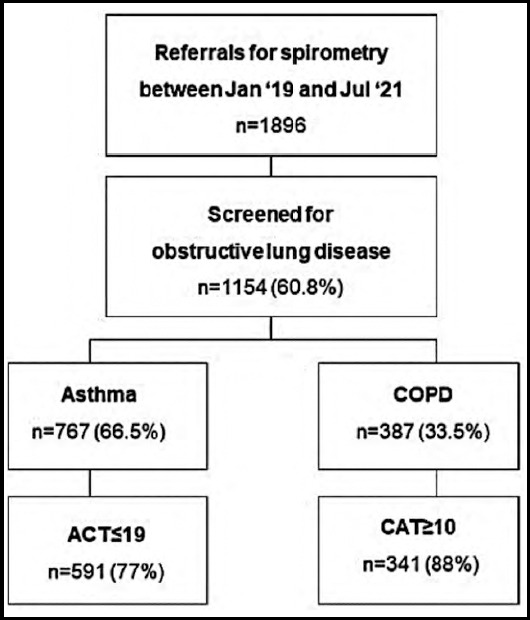
Obstructive lung disease and severity.

Baseline spirometry was offered to all suspected OLD cases (n=1154). A diagnosis of OLD was based on spirometry in 845 cases (73.2%) whilst clinical diagnosis alone accounted for 309 (26.8%). Airflow obstruction ranged from severe to very severe intensity in 42.6% of COPD and 32.5% of asthma cases. Using mMRC, 45.2% of OLD patients scored two or above. CAT scores in COPD showed 88% had poor symptom control whilst ACT scores demonstrated uncontrolled asthmatic disease in 77% (Fig.3).

Staging for COPD patients showed 11.6% at GOLD stage A (less risk, less symptoms), 31.3% stage B (less risk, more symptoms), 1.3% stage C (high risk, less symptoms) and 55.8% at stage D (high risk, more symptoms). Two or more exacerbations requiring increased therapy (oral steroids and/or antibiotics or increased reliever medication) were seen in 42% of OLD patients.

### Management:

Of 1129 OLD patients, 54% had inhaled therapy prescribed. This included combined inhaled corticosteroid and long-acting beta 2-agonists (ICS/LABA, 39.3%), short-acting beta2-agonists (SABA, 28.2%) and long-acting antimuscarinics (10.2%). Montelukast (31.4%) was prescribed as an add-on oral therapy. Inhaler technique was taught in 66.5%. Those with mMRC score of two and above (n=234) were referred for PR in 96.2% of cases. Advice on tobacco cessation was delivered to 61.1% of all current users (n=229).

## DISCUSSION

Integration of the OLD program into Family Medicine through training and formal diagnostics showed that (1) e-curriculums enable improved HCP knowledge of OLD, (2) asthma was more common than COPD, was associated with more co-morbidities and was predominant in females. In this population, a high prevalence of biomass exposure and both smoking and smokeless tobacco use was noted in both asthma and COPD. Spirometry revealed advanced disease in over half the patients with OLD.

The OLD program is an integrated care program where CRDs, namely asthma and COPD, are diagnosed and managed in primary care and to our knowledge is the first of its kind in Pakistan. Implementation strategies for interventions in lung health in LMICs have been suggested by Brakema and colleagues.^6^ They collated and interpreted implementation challenges to provide a broad toolkit for LMIC. Similar strategies are emphasized in the WHO package of essential noncommunicable disease interventions for primary care, aiming to bridge the gaps in universal health coverage for those impacted with NCDs.^17^ In line with this, we aimed to identify and address local challenges in integration, by gaining stakeholder and resource support. Primary care facilities at IHHN were conducive to program integration with HCP training, thereby providing an ideal platform for further OLD implementation.

Through our e-learning platform, HCPs were introduced to updated, evidence-based learning material covering all aspects of OLD in a flexible and accessible manner. In Pakistan, a precedent for e-learning was reported by The Aga Khan University. Their massive open online course on Drug Discovery was well received by participants.^18^ Several postgraduate online courses in India and other LMICs were reported by Gupta and colleagues.^19^ Meanwhile, in Latin America, a tobacco cessation module was successfully completed by hospital-based HCPs online and the value of sharing resources amongst culturally similar countries emphasized.^20^ All highlighted the need for strong organizational and resource support for success in e-learning. The value of digital medical education is being emphasized both in LMICs^21^ and worldwide.^22^

In our experience, limitations to training were seen as HCP turnover interrupted the e-curriculum format and created lag time. Internet access was variable. Training time was often compromised by other clinical activity during the work day, delaying completion. Importantly, whilst pre- and post-test evaluations demonstrate improved learning, we cannot conclude a clear link with improved diagnosis of OLD within the constraints of this work. This is similar to the e-learning strategy reported in the UK, teaching fundamentals of prescription writing and causes of prescription errors. Whilst pre and post course assessments done on general practitioners and pharmacists demonstrated scores increased by 17.4%, the impact on patient outcomes could not be established.^23^

Our data collection methods were online and easily accessible. This information can be interrogated to provide important epidemiological data on these poorly documented NCDs. Notably the population selected for confirmation of OLD were likely suspects of disease, so true incidence and prevalence figures cannot be reliably interpretated. Patients with OLD are more likely to abuse tobacco, we identified 34% were smokers of our patient, higher than the national average.^12^ The strength of the program is the high detection of OLD based on clinical screening. As the program gains traction, a database of clinical findings, diagnostics and management strategies can be reported. The method of data collection also aides monitoring and evaluation, simplifying performance of clinical audit and ensuring clinical governance. Similar to e-learning, limitations involve consistent internet access and time for training and data entry.

Amongst patients who were screened for OLD, 16.2% were unable to perform spirometry in their first visit. Technicians performing spirometry have an important role in appropriate coaching but factors such as worsened breathlessness, coughing or communication issues can still limit outcomes. Where spirometry provides a gold standard for diagnosis, its absence relies on clinical judgement and we noted 26.8% of diagnosed OLD patients had no spirometry performed. Meanwhile, despite careful HCP training, we found inadequate inhaler therapy prescription in some patients. Our program could not evaluate the causes of this, which may include unaffordability, lack of compliance or need for remedial training of HCPs. Advice on tobacco cessation was missing in 59% eligible cases, possibly demonstrating a gap in training.

Spirometry completely halted in April 2020 in view of the COVID pandemic, as it is an aerosol generating procedure likely to spread disease. This limitation inevitably impacted program performance and data collection. Since August 2020, services were restarted with careful verbal screening by technical staff who were otherwise in full personal protective equipment and were relocated to ventilated areas. A pragmatic approach to continue services, as done worldwide, was therefore adapted.

The programmatic requirements of extensive data collection and highly trained resources for each patient may serve as a limitation by proving expensive and difficult to further integrate. However, the program provides an evidence-based package, impacting both the patient and their health ecosystem. Patients may have improved self-awareness and slower decline in lung function. Meanwhile, the health system may have a reduced burden of emergency visits and hospitalizations. A complete cost-benefit analysis is warranted to evaluate this further. Other potential areas of development include development of home-based support both in the medical and non-medical sectors, completing the cycle and becoming truly integrated practice units and integrated care programs.

## CONCLUSION

The Obstructive Lung Disease program has shown itself to be a useful primer for establishing a sustainable and scalable integrated care program within primary care. It has used innovative training models, dealt with implementation challenges and provided learning opportunities on how to develop further. Providing evidence-based care for common NCDs begins to address the Sustainable Development Goals (good health and well-being and quality education in LMICs) within Pakistan. Further expansion is planned to protect the health of our communities.

### Authors’ Contribution:

**SS:** Conception and design, program development, drafting the manuscript, revision for accuracy an is responsible for integrity of the study.

**MS:** Educational module development, data interpretation, manuscript drafting, revision for accuracy.

**RA:** Design of data collection tools and acquisition of data, computations and analysis, manuscript drafting, revision for accuracy.
